# The Relationship Between Short-Term Surrogate Endpoint Indicators and mPFS and mOS in Clinical Trials of Malignant Tumors: A Case Study of Approved Molecular Targeted Drugs for Non-Small-Cell Lung Cancer in China

**DOI:** 10.3389/fphar.2022.862640

**Published:** 2022-03-16

**Authors:** Mingjun Rui, Zijing Wang, Zhengyang Fei, Yao Wu, Yingcheng Wang, Lei Sun, Ye Shang, Hongchao Li

**Affiliations:** ^1^ School of International Pharmaceutical Business, China Pharmaceutical University, Nanjing, China; ^2^ Center for Pharmacoeconomics and Outcomes Research, China Pharmaceutical University, Nanjing, China

**Keywords:** malignant tumor, short-term surrogate endpoint, median progression-free survival, median overall survival, non-small-cell lung cancer

## Abstract

**Objective:** Due to the initiation of the priority review program in China, many antitumor drugs have been approved for marketing based on phase II clinical trials and short-term surrogate endpoint indicators. This study used approved targeted drugs for the treatment of non-small-cell lung cancer (NSCLC) in China as an example to evaluate the association between short-term surrogate endpoints [objective response rate (ORR) and disease control rate (DCR)] and median progression-free survival (mPFS) and median overall survival (mOS).

**Methods:** Five databases, i.e., MEDLINE, Embase, Cochrane Library, China National Knowledge Infrastructure (CNKI), and Wanfang Data were searched, for phase II or phase III clinical trials of all molecular targeted drugs that have been marketed in China for the treatment of NSCLC. After screening the literature and extracting information, both univariate and multivariate linear regression were performed on the short-term surrogate indicators and mPFS and mOS to explore the relationship.

**Results:** A total of 63 studies were included (25 studies with only ORR, DCR, and mPFS and 39 studies with ORR, DCR, mPFS, and mOS). In terms of the targeted drugs for the treatment of NSCLC, in addition to the good but not excellent linear relationship between DCR and mOS (0.4 < R^2^_adj_ = 0.5653 < 0.6), all other short-term surrogate endpoint indicators had excellent linear relationships with mPFS and mOS (R^2^_adj_≥0.6), while mPFS and mOS had the most excellent linear relationships (R^2^_adj_ = 0.8036).

**Conclusion:** For targeted drugs for the treatment of NSCLC, short-term surrogate endpoint indicators such as ORR and DCR may be reliable surrogate indicators for mPFS and mOS. However, whether short-term surrogate endpoint indicators can be used to predict final endpoints remains to be verified.

## Introduction

Malignant tumors are a high-risk factor for death and severely hinder increases in the average life expectancy of the population (1). They are the leading cause of death in the urban population. In 2019, approximately 25.73% of urban population deaths in China were caused by malignant tumors, with a mortality rate of approximately 161.56/100,000 people (2). In 2018, there were 3.804 million new cases of malignant tumors in China, accounting for more than 20% of the global cases. The incidence of malignant tumors was 278.07 per 100,000 people, and the mortality rate was 167.89 per 100,000 people (Ma and Yu, 2020). Malignant tumors seriously threaten the lives and health of people. From the perspective of disease burden, malignant tumors have caused a substantial loss of disability-adjusted life years (DALYs). Studies have shown that (3) the proportion of DALYs caused by trachea, bronchus, and lung cancers was 4.1% of the total DALYs, ranking fourth only after stroke (11.9%), ischemic heart disease (8.1%), and chronic obstructive pulmonary disease (5.5%). From the economic burden perspective, the average medical costs for malignant tumor patients are increasing year by year. In 2005, the average cost of a single hospitalization for discharged patients in China was 10,777 yuan (RMB), increasing to 13,322 yuan in 2011, 15,672 yuan in 2013, and 17,567 yuan in 2016 (Wei-jing and xiao-lu, 2019).

To increase patients’ accessibility to new drugs and to improve the quality of life, the National Medical Products Administration (NMPA) in China launched a priority review program to allow more innovative drugs to be approved as soon as possible to bring patients with malignant tumors benefits. The NMPA priority review processes mainly include three policies: one review process for breakthrough therapeutic drugs, one review process for the conditional approval of drugs for marketing, and one priority review process for drug marketing authorization (Adminstration, 2020). The priority review program greatly shortens the time to market for some new anticancer drugs which often focus on the rare targets, and many of them do not have abundant clinical data based on Chinese patients. Many of these clinical studies are often single-arm with a small sample size and short follow-ups, and even primary endpoint indicators such as progression-free survival (PFS) and overall survival (OS) were not reported.

Table 1 summarized the reported status of clinical trial indicators for anticancer drugs approved in China from 2017 to November 2021. An increasing number of drugs were approved using only short-term surrogate endpoint indicators. Among them, only 16 new drugs reported both PFS and OS data. However, the lack of primary endpoint indicators causes challenges in reliably determining the safety and efficacy of anticancer drugs and, likewise, poses a significant challenge for economic evaluations. In the economic evaluation of anticancer drugs, the partitioned survival model (PSM) and the Markov model are most popular model types (Rui et al., 2021). The construction of both the PSM and the Markov model requires the support of mature PFS and OS data (6). Therefore, when only short-term surrogate endpoint indicators available, it is worth investigating whether there is a significant relationship between such indicators and primary endpoint indicators.

**TABLE 1:** Summary of clinical endpoints of new anti-cancer drugs approved from January 2017 to November 2021.

Drug	Approved year	Disease	mPFS (month)	mOS (month)	ORR	mDOR (month)	DCR
PFS and OS							
Vemurafenib	2017	Melanoma	8.3	13.5	52%	—	46%
Regorafenib	2017	mCRC	1.9	6.4	1%	—	41%
		GIST					
Bevacizumab	2017	mCRC	4.2	9.3	41.20%	8.1	—
		NSCLC					
Anlotinib	2018	NSCLC	5.37	9.46	9.18%	—	80.95%
Pembrolizumab	2018	Melanoma	2.8	12.1	16.70%	8.4	38.20%
Lenvatinib	2018	HCC	7.4	13.6	24.10%	—	—
Fruquintinib	2018	mCRC	3.7	9.3	4.70%	5.6	62.20%
Bendamustine	2018	Lymphoma	18.6	74%	—	16.5	—
Eribulin	2019	Breast Cancer	2.8	13.4	30.70%	—	—
FTD/TPI	2019	mCRC	2	7.8	—	—	—
Pralatrexate	2020	T cell Lymphoma	3.6	14.5	39%	10.1	—
Atezolizumab	2020	SCLC	5.2	12.3	60.20%	4.2	—
Savolitinib	2021	NSCLC	6.8	12.5	42.90%	8.3	82.90%
Utidelone	2021	Breast Cancer	8.44	16.13	40.40%	7.59	53.90%
Donafenib	2021	HCC	12.1	3.7	4.60%	—	30.8%
Carfilzomib	2021	Myeloma	5.6	16.6	35.80%	—	—
PFS and ORR/DCR/DOR							
Afatinib	2017	NSCLC	11.01	—	67.80%	9.72	92.60%
Osimertinib	2017	NSCLC	9.7	—	62.70%	9.9	88.60%
Ibrutinib	2017	Lymphoma	13.9	—	67.60%	14.9	—
Pazopanib	2017	RCC	11.1	—	30%	—	—
Erlotinib	2017	NSCLC	10.4	—	65.10%	—	—
Ixazomib	2018	Myeloma	19.6	—	78.30%	—	—
Pyrotinib	2018	Breast Cancer	18.1	—	78.50%	16.7	—
Alectinib	2018	NSCLC	34.8	—	83%	—	—
Olaparib	2018	Ovarian Cancer/PPC	19.1	—	19.40%	—	81.00%
Toripalimab	2018	Melanoma	3.6	—	17.30%	—	57.50%
Sintilimab	2018	HL	15.4	—	84%	—	100%
Dacomitinib	2019	NSCLC	14.7	—	75%	14.8	—
Denosumab	2019	GCT	—	—	25%	—	—
Camrelizumab	2019	HL	11.3	—	80.30%	—	98.50%
Almonertinib	2020	NSCLC	12.3	—	68.90%	12.4	93.40%
Surufatinib	2020	NEN	9.2	—	10.30%	5.6	86.50%
Inetetamab	2020	Breast Cancer	9.12	—	46.70%	—	79.72%
Ensartinib	2020	NSCLC	11.2	—	52.60%	—	87.80%
Ripretinib	2021	GIST	6.3	—	11.80%	—	—
Furmonertinib	2021	NSCLC	7.6	—	73.60%	—	—
Donafenib	2021	HCC	12.1	3.7	4.60%	—	30.8%
Carfilzomib	2021	Myeloma	5.6	16.6	35.80%	—	—
ORR/mDOR/DCR							
Denosumab	2019	GCT	—	—	25%	—	—
Tislelizumab	2019	HL	—	—	76.90%	—	90.80%
Rituximab	2019	Lymphoma	—	—	94.10%	—	—
Zanubrutinib	2020	T cell Lymphoma	—	—	83.70%	19.5	—
Orelabrutinib	2020	Leukemia/SLL	—	—	73.80%	—	—
Neratinib	2020	Breast Cancer	—	—	32.80%	—	—
Fluzoparib	2020	PC	—	—	64.10%	—	—
Venetoclax	2020	AML	—	—	—	5.5	—
Pamiparib	2021	Ovarian Cancer/PFTC/PPC	—	—	68.30%	13.8	—
Avapritinib	2021	GIST	—	—	62.50%	—	—
Pralsetinib	2021	NSCLC	—	—	65%	—	93%
Daratumumab	2021	Myeloma	—	—	83%	—	34.3%
Penpulimab	2021	HL	—	—	84.70%	—	94.10%
Zimberelimab	2021	HL	—	—	90.48%	—	96.43%

PFS, progression-free survival; OS, overall survival; DOR, duration of response; mPFS, median progression-free survival; mOS, median overall survival; ORR, overall response rate; mDOR, median duration of response; DCR, disease control rate;/: not erported; mCRC, metastatic colorectal cancer; GIST, gastrointestinal stromal tumor; NSCLC, non-small cell lung cancer; HCC, hepatocellular carcinoma; SCLC, small cell lung cancer; RCC, renal cell carcinoma; PPC, primary peritoneal carcinoma; HL, hodgkin lymphoma; GCT, giant cell tumor of bone; NEN, neuroendocrine neoplasm; SLL, small lymphocytic lymphoma; PC, peritoneal carcinomatosis; AML, acute myeloid leukemia; PFTC, primary fallopian tube carcinoma; FTD/TPI, trifluridine/tipiracil.

To explore this issue, this study will use clinical trials related to targeted therapy for the treatment of NSCLC approved in China as an example to explore the correlation between short-term surrogate endpoint indicators and primary endpoint indicators.

## Materials and Methods

### Literature Search Strategy

Chinese and English databases and platforms were searched for Phase II or phase III clinical trials of molecular targeted drugs for the treatment of NSCLC. The Chinese search included China National Knowledge Infrastructure (CNKI) and Wanfang Data. The English search included MEDLINE through the PubMed search platform, Embase, and the Cochrane Library. The date ranges for the searches were from the establishment of the databases to 20 March 2021. The searches were based on a combination of subject headings and free-text. Chinese search terms and English search terms included non-small-cell lung cancer and clinical trial, among other terms. The English search strategy was shown in the supplementary materials.

### Inclusion and Exclusion Criteria

The inclusion criteria for this study were as follows: (Bray et al., 2021) phase II or phase III clinical trials, including single-arm clinical trials and placebo-controlled clinical trials (Ma and Yu, 2020); patients diagnosed with NSCLC by laboratory tests, imaging examinations and clinical signs and symptoms (Wei-jing and xiao-lu, 2019); intervention measures that included molecular targeted drugs for the treatment of NSCLC approved for marketing in China as of March 2021, including gefitinib, erlotinib, icotinib, crizotinib, dacomitinib, afatinib, osimertinib, almonertinib, alectinib, ceritinib, brigatinib, lorlatinib, selpercatinib, entrectinib, dabrafenib + trametinib, erlotinib + linsitinib, erlotinib + pazotinib, erlotinib + sorafenib, and anlotinib (Adminstration, 20202020); short-term surrogate endpoint indicators included ORR or DCR; and (Rui et al., 2021) primary endpoint indicators included median progression-free survival (mPFS) and median overall survival (mOS). The exclusion criteria for this study were as follows: (Bray et al., 2021): duplicate literature (Ma and Yu, 2020); non-Chinese or non-English literature (Wei-jing and xiao-lu, 2019); conference abstract (Adminstration, 20202020); trials other than phase II or phase III clinical trials (Rui et al., 2021); no simultaneous reporting of DCR, ORR, and mPFS; and (Coyle and Coyle, 2014) intervention measures that included molecular targeted drugs combined with other types of therapeutic measures.

### Literature Screening and Data Extraction

Two researchers independently screened the literature, extracted the data, and cross-checked the data. Disagreements were resolved through consultation with a third party. Data extraction mainly included ① basic characteristics of the included studies (title, authors, year, etc.); ② sample size of each group; ③ treatment measures and their usage and dosage; ④ key elements of bias risk assessments; and ⑤ endpoint indicators (ORR, DCR, mPFS and mOS).

### Quality of the Included Studies

Two investigators independently conducted quality evaluations of the included studies and cross-checked the results. For randomized controlled clinical trials (RCTs), the quality of the included studies was evaluated using the risk of bias assessment tool for RCTs recommended by the Cochrane Manual (Higgins et al., 2011). The Newcastle–Ottawa scale (NOS), recommended by the Cochrane Non-Randomized Studies Methods Group (NRSMG), was used to evaluate the quality of single-arm clinical trials (Margulis et al., 2014).

### Data Processing

This study used STATA 15.1 to perform both univariate and multivariate linear regression analysis of the relationship between DCR and ORR and mPFS, as well as the relationship between DCR and ORR and mOS. In the case of a poor linear relationship between the shot-term surrogate endpoint indicators and the primary endpoint indicators, ln transformation was performed on the short-term surrogate endpoint indicators to explore the linear relationship between ln (short-term surrogate endpoint indicators) and the primary endpoint indicators. For the different dosage, medication or duration included in the analysis, the treatments were categorized for inclusion in multivariate regression analysis. In addition, some studies showed that the OS is largely affected by the number of previous treatment lines, which means that patients received more lines of treatments often have a worse prognosis (Gisselbrecht et al., 2010; Rule et al., 2017). Therefore, the subgroup analyses were performed for first-line treatment and second-line or post-second-line treatment based on the number of treatment lines in the univariate linear regression analysis to separate patients with different treatment lines to reduce heterogeneity. The scatter plots for DCR and ORR vs. mPFS and mOS were plotted using Microsoft Excel. Adjusted goodness-of-fit R^2^_adj_ was used to evaluate the degree of fit of the model. According to Lassere et al. (Lassere et al., 2012), R^2^_adj_≥0.6 indicates excellent goodness-of-fit, R^2^_adj_≥0.4 indicates good goodness-of-fit, R^2^_adj_≥0.2 indicates fair goodness-of-fit, and R^2^_adj_<0.2 indicates poor goodness-of-fit.

## Results

### Literature Screening Results

**FIGURE 1:**
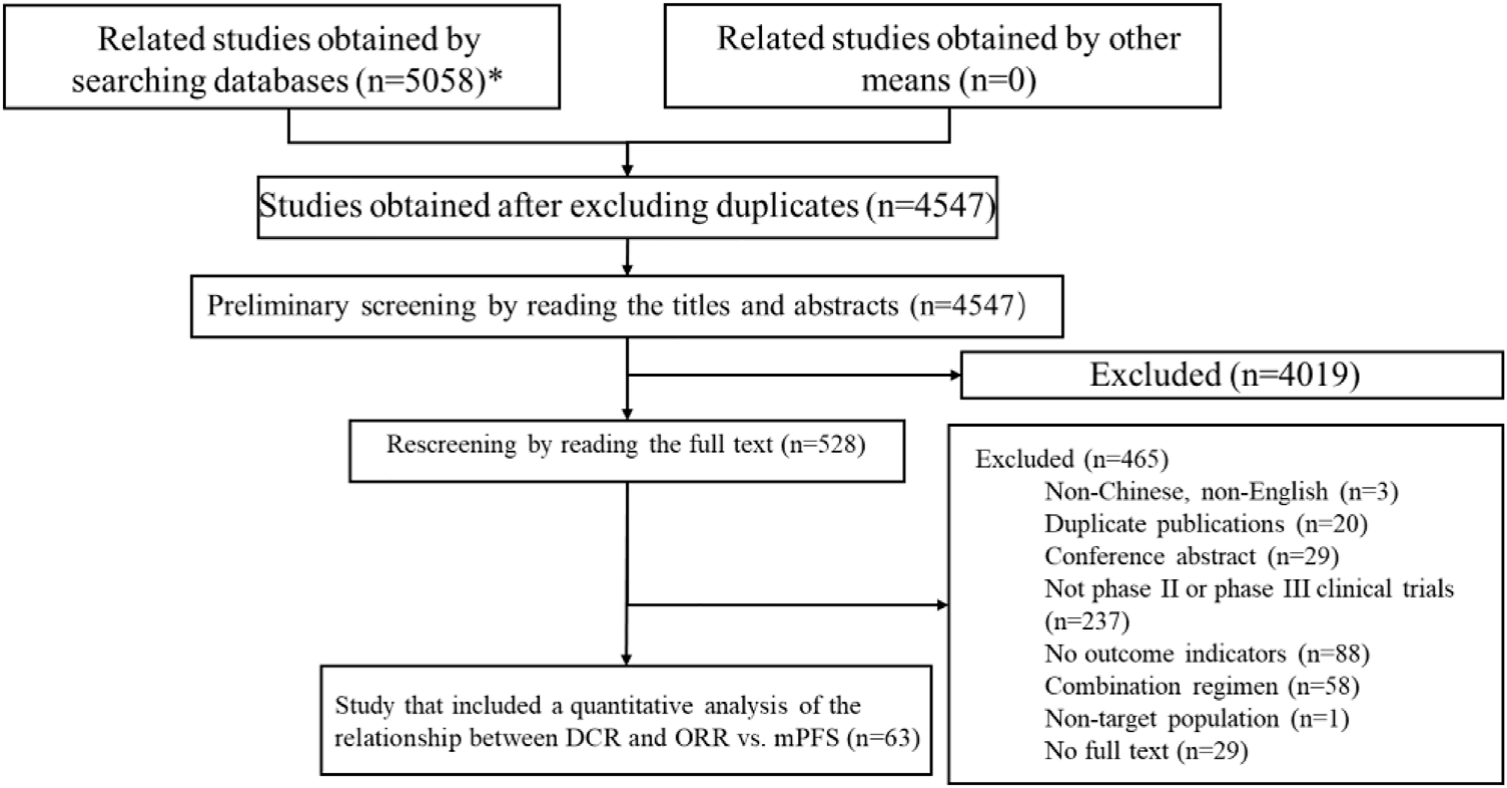
Flowchart of literature screening.

A total of 5,058 articles were obtained in the preliminary searches, and a total of 4,547 articles were included in the preliminary screening after excluding duplicates. After reading the titles and abstracts, 4,019 papers were excluded, and 528 papers were included in the full-text rescreening. After reading the full text of the 528 papers, 63 articles were included in the final sample for the quantitative analysis of DCR, ORR, mPFS and mOS. The literature screening process is shown in Figure 1.

### Results of the Data Extraction From the Included Studies

Among the 63 included studies, 25 (Inoue et al., 2006; Wen et al., 2007; Kim et al., 2012; Yamada et al., 2013; Hu et al., 2015; Yoshimura et al., 2015; Park et al., 2016; Yang et al., 2017a; Leighl et al., 2017; Peters et al., 2017; Han et al., 2018a; Akamatsu et al., 2018; Wu et al., 2018a; Nie et al., 2018; Yang et al., 2018; Zhou et al., 2018; Deng et al., 2019; Landi et al., 2019; Michels et al., 2019; Ohe et al., 2019; Saito et al., 2019; Yang et al., 2019; Yokoyama et al., 2019; Yang et al., 2020; de Marinis et al., 2021) only reported ORR, DCR, and mPFS, and 39 (Guan et al., 2005; Zhou et al., 2009; Gaafar et al., 2011; Lee et al., 2011; Spigel et al., 2011; Ciuleanu et al., 2012; Deng et al., 2012; Miller et al., 2012; Pallis et al., 2012; Pérol et al., 2012; Ramalingam et al., 2012; Shi et al., 2013; Wu et al., 2013; Ramalingam et al., 2014; Choi et al., 2015; Juan et al., 2015; Wu et al., 2015; An et al., 2016; Ding-guo and Xiu-li, 2016; Neal et al., 2016; Yang et al., 2017b; Han et al., 2017; Ikezawa et al., 2017; Miyawaki et al., 2017; Han et al., 2018b; Wu et al., 2018b; Garon et al., 2018; Kiura et al., 2018; Spigel et al., 2018; Uchibori et al., 2018; Arrieta et al., 2019; Landi et al., 2019; Xu et al., 2019; Ahn et al., 2020; Eide et al., 2020; Gadgeel et al., 2020; Goldman et al., 2020; Huber et al., 2020; Scagliotti et al., 2020) reported ORR, DCR, mPFS, and mOS concurrently. One paper (19) grouped the patients for whom ORR, DCR, and mPFS were reported and the patients for whom ORR, DCR, mPFS and mOS were reported concurrently. Basic information and the ORR, DCR, mPFS and mOS values reported in the included studies are provided in Tables 2, 3.

**TABLE 2:** Overview of general characteristics of studies reporting ORR, DCR, and mPFS.

References	Population	Brain metastasis	Line	Arm	Sample size	Treatment	Dosage	DCR (%)	ORR (%)	mPFS (month)
de Marinis et al. (2021)	Locally advanced or metastatic EGFR mutation-positive NSCLC	No	First	Single	479	AfatiTnib	40 mg/qd	85.80	49.20	13.40
Yang et al. (2019)	Locally advanced or metastatic NSCLC	No	1st	Double	17	Osimertinib	80 mg/qd	100.00	80.00	19.30
Ohe et al (2019)	Advanced or metastatic NSCLC	No	First	Double	65	Osimertinib	80 mg/qd	96.90	75.40	19.10
Ohe et al (2019)	Advanced or metastatic NSCLC	No	First	Double	55	Gefitinib	250 mg/qd	96.40	76.40	13.80
Deng et al. (2019)	Advanced or metastatic NSCLC	Yes	First + Second	Single	47	Crizotinib	-	93.60	61.70	19.00
Yokoyama et al. (2019)	Advanced NSCLC	No	First	Single	46	Afatinib	20mg/qd	93.20	81.80	15.20
Michels et al. (2019)	Metastatic ROS1-rearranged NSCLC	No	First	Single	30	Crizotinib	250 md/bid	83.30	73.00	20.00
Nie et al. (2018)	EGFR T790M mutated NSCLC	Yes	Third	Double	73	Osimertinib	80 mg/qd	87.70	61.60	10.20
Akamatsu et al. (2018)	EGFR T790M mutated NSCLC	Yes	First	Double	41	Osimertinib	80 mg/qd	95.10	70.70	10.10
Wu et al. (2018a)	Advanced NSCLC	Yes	First	Double	75	Osimertinib	80 mg/qd	40.00	87.00	5.50
Wu et al. (2018b)	Advanced NSCLC	Yes	First	Double	30	Osimertinib	80 mg/qd	93.00	70.00	5.60
Saito et al. (2019)	EGFR-positive advanced NSCLC	Yes	First	Double	112	Erlotinib	150 mg/qd	96.00	67.00	13.30
Han et al. (2018a)	Advanced NSCLC	No	3rd	Double	60	Anlotinib	12 mg/qd	83.30	10.00	4.80
Peters et al. (2017)	ALK-positive NSCLC	Yes	First	Double	151	Ceritinib	500 mg/qd	91.00	75.00	11.10
Landi et al. (2019)	ROS1-rearranged NSCLC	No	Second	Single	33	Crizotinib	250 mg/bid	85.00	65.00	22.80
Yang et al. (2018)	Advanced NSCLC	No	First	Double	35	Gefitinib	250 mg/qd	80.00	57.10	8.40
Zhou et al. (2018)	ALK-positive advanced NSCLC	Yes	First	Double	31	Crizotinib	-	100.00	78.10	16.10
Kim et al. (2012)	Advanced NSCLC	No	Second	Double	48	Erlotinib	150 mg/qd	66.70	39.60	3.10
Kim et al. (2012)	Advanced NSCLC	No	Second	Double	48	Gefitinib	250 mg/qd	72.90	47.90	4.90
Inoue et al. (2006)	Advanced NSCLC	No	First	Single	16	Gefitinib	250 mg/qd	88.00	75.00	9.70
Leighl et al. (2017)	Advanced NSCLC	No	First	Double	44	Erlotinib	150 mg/qd	77.30	47.70	8.40
Leighl et al. (2017)	Advanced NSCLC	No	First	Double	44	Linsitinib + Erlotinib	Linsitinib: 150 mg/bid	95.50	75.00	12.40
							Erlotinib: 150 mg/qd			
Wen et al. (2007)	Advanced NSCLC	No	Second	Double	20	Gefitinib	250 mg/qd	85.00	75.00	9.50
Wen et al. (2007)	Advanced NSCLC	No	Second	Double	30	Gefitinib	250 mg/qd	43.00	20.00	3.70
Yamada et al. (2013)	EGFR T790M positive advanced NSCLC	No	Second	Single	14	Erlotinib	150 mg/qd	80.80	53.80	9.30
Yang et al. (2020)	Crizotinib-resistant, ALK-positive NSCLC	Yes	Second	Single	160	Ensartinib	225 mg/qd	93.00	52.00	9.60
Yang et al. (2017a)	T790M-Positive Advanced NSCLC	Yes	Second + Third	Single	201	Osimertinib	80 mg/dq	90.00	62.00	12.30
Park et al. (2016)	EGFR mutation-positive NSCLC	Yes	First	Double	160	Afatinib	40 mg/d	91.00	70.00	11.00
Hu et al. (2015)	Advanced NSCLC	No	Second	Single	124	Icotinib	125 mg/tid	67.70	25.80	5.00
Yoshimura et al. (2015)	EGFR mutated, advanced non-squamous NSCLC	No	First	Single	26	Pemetrexed + Gefitinib	250 mg/q3w	96.20	84.60	18.00

DCR, disease control rate; ORR, overall response rate; mPFS, median progression-free survival; NSCLC, non-small cell lung cancer; EGFR-TKI(s), EGFR tyrosine kinase inhibitors; qd, once a day; bid, twice a day; tid, three times a day; d, day; q3w, once every 3 weeks;—, not mentioned.

**TABLE 3:** Overview of general characteristics of studies reporting ORR, DCR, mPFS, and OS.

References	Population	Brain metastasis	Line	Arm	Sample size	Treatment	Dosage	DCR (%)	ORR (%)	mPFS (month)	mOS (month)
Yang et al. (2017b)	Locally advanced or metastatic ALK-positive NSCLC	No	First	Single	225	Alectinib	600 md/bid	78.80	51.30	8.30	26.00
Wu et al. (2018a)	Advanced NSCLC	No	First	Double	242	Afatinib	50 mg/qd	92.20	66.80	11.00	31.60
Uchibori et al. (2018)	Advanced NSCLC	No	First	Single	33	Gefitinib	250mg/qd	81.81	22.90	6.70	24.30
Ikezawa et al. (2019)	EGFR mutated advanced NSCLC	Yes	Third + Fourth	Double	19	Erlotinib	150 mg/qd	42.10	15.80	1.60	8.00
Garon et al. (2018)	Advanced NSCLC	No	First	Double	33	Erlotinib	150 mg/qd	79.10	12.10	3.50	9.50
Kiura et al. (2018)	ALK-rearranged advanced NSCLC	Yes	First	Double	11	Ceritinib	750 mg/qd	90.90	54.50	9.80	23.90
Cheng et al. (2021)	Advanced NSCLC	Yes	Third	Double	27	Anlotinib	12 mg/qd	82.89	9.65	4.80	10.70
Cheng et al. (2021)	Advanced squamous NSCLC	Yes	Third	Double	36	Anlotinib	12 mg/qd	71.70	7.55	5.50	9.60
Miyawaki et al. (2017)	EGFR mutated advanced NSCLC	Yes	Second + Third	Single	13	Erlotinib	150 mg/qd	69.20	53.80	7.80	25.10
Miyawaki et al. (2017)	wild-type EGFR advanced NSCLC	Yes	Second + Third	Single	22	Erlotinib	150 mg/qd	31.80	9.10	2.10	14.9
Landi et al. (2019)	MET-deregulated or ROS1-rearranged NSCLC	No	Second	Single	37	Crizotinib	250 mg/bid	69.00	27.00	4.40	5.4
Pérol et al. (2012)	Advanced NSCLC	No	Second	Multi	155	Erlotinib	150 mg/qd	35.64	10.89	2.90	11.40
Spigel et al. (2011)	Advanced NSCLC	No	Second	Double	111	Erlotinib + Sorafenib	Sorafenib: 400 mg/bidErlotinib: 150 mg/qd	54.00	8.10	3.38	7.62
Spigel et al. (2011)	Advanced NSCLC	No	Second	Double	55	Erlotinib	150 mg/qd	38.20	10.90	1.94	7.23
Deng et al. (2012)	Advanced NSCLC	No	Second	Single	40	Gefitinib	250 mg/qd	92.50	62.50	13.00	20.00
Lee et al. (2011)	Advanced or metastatic NSCLC	Yes	First	Single	24	Erlotinib	100 mg/qd	25.00	21.00	1.50	3.20
Spigel et al. (2018)	Advanced NSCLC	No	Second	Double	127	Pazopanib + Erlotinib	Pazopanib: 600 mg/qdErlotinib: 150 mg/qd	44.00	10.00	2.60	6.90
Spigel et al. (2018)	Advanced NSCLC	No	Second	Double	65	Erlotinib	150 mg/qd	34.00	5.00	1.80	7.00
Ciuleanu et al. (2012)	Advanced or metastatic NSCLC	Yes	Second	Double	203	Erlotinib	150 mg/qd	34.50	7.90	1.47	5.30
Arrieta et al. (2019)	Advanced NSCLC	No	First	Double	70	EGFR-TKIs	-	91.40	54.30	9.90	17.50
Han et al. (2018a)	Advanced NSCLC	No	Third	Double	296	Anlotinib	12 mg/qd	81.00	9.20	5.40	9.60
Xu et al. (2019)	Advanced NSCLC	No	First	Double	89	Icotinib	125 mg/tid	79.80	64.00	10.00	34.00
Han et al. (2017)	Advanced NSCLC	No	First	Multi	41	Gefitinib	250 mg/qd	97.60	65.90	11.90	25.80
Zhou et al. (2009)	Advanced NSCLC	—	Second	Single	112	Erlotinib	150 mg/qd	76.80	35.70	6.30	12.30
Miller et al. (2012)	Advanced NSCLC	No	Second + Third	Double	390	Afatinib	50 mg/qd	58.00	7.00	3.30	10.80
Pallis et al. (2012)	Advanced or metastatic NSCLC	Yes	First	Single	49	Erlotinib	150 mg/qd	69.40	24.50	6.70	11.50
Gaafar et al. (2011)	Advanced or metastatic NSCLC	Yes	Second	Double	86	Gefitinib	250 mg/qd	79.10	11.60	4.10	10.90
Guan et al. (2005)	Advanced or metastatic NSCLC	Yes	Second + Third	Single	153	Gefitinib	250 mg/qd	54.10	27.00	3.23	10.00
Ramalingam et al. (2012)	Advanced NSCLC	No	First	Double	94	Dacomitinib	45 mg/qd	29.80	17.00	2.86	9.53
Ramalingam et al. (2012)	Advanced NSCLC	No	First	Double	94	Erlotinib	150 mg/qd	14.90	5.30	1.91	7.44
Ahn et al. (2020)	EGFR T790M positive advanced NSCLC	Yes	Second	Single	62	Osimertinib	80 mg/qd	95.00	74.00	10.90	29.20
Eide et al. (2020)	EGFR mutated advanced NSCLC	No	Second	Single	199	Osimertinib	80 mg/qd	83.00	48.00	8.90	17.90
Goldman et al. (2020)	Advanced NSCLC	No	Second	Double	183	Erlotinib	150 mg/qd	31.70	2.70	1.90	7.80
Huber et al. (2020)	Crizotinib-refractory ALK positive NSCLC	Yes	Second	Double	112	Brigatinib	90mg/qd	78.00	51.00	9.20	29.50
Ramalingam et al. (2014)	Advanced NSCLC	No	Second	Double	439	Dacomitinib	45 mg/qd	48.97	11.39	2.60	8.10
Ramalingam et al. (2014)	Advanced NSCLC	No	Second	Double	439	Erlotinib	150 mg/qd	49.66	8.20	2.60	8.50
Scagliotti et al. (2020)	Advanced NSCLC	No	First	Double	70	Erlotinib	150 mg/qd	98.60	65.70	9.50	25.40
Shi et al. (2013)	Advanced NSCLC	No	Second	Double	199	Icotinib	125 mg/tid	75.40	27.60	4.60	13.30
Shi et al. (2013)	Advanced NSCLC	No	Second	Double	196	Gefitinib	250 mg/qd	74.90	27.20	3.40	13.90
An et al. (2016)	EGFR mutated non-squamous NSCLC	No	First	Double	45	Gefitinib	250 mg/q3w	86.67	73.33	14.00	32.00
Neal et al. (2016)	EGFR wild-type advanced NSCLC	Yes	Second + Third	Triple	42	Erlotinib	150 mg	18.40	3.00	1.80	5.10
Ding-guo and Xiu-li (2016)	Advanced NSCLC	—	Second	Double	50	Gefitinib	250 mg/d	64.00	24.00	5.20	7.90
Choi et al. (2015)	Chemotherapy-naïve NSCLC	No	First	Double	43	Paclitaxel + Carboplatin + Gefitinib	250 mg/q3w	74.40	41.90	4.10	9.30
Wu et al. (2015)	Advanced EGFR mutation-positive NSCLC	No	First	Double	110	Erlotinib	150 mg/qd	89.10	62.70	11.00	26.30
Juan et al. (2015)	Advanced NSCLC	—	Second	Double	33	Docetaxel + Erlotinib	150 mg/qd	52.00	3.00	3.00	7.50
Wu et al. (2015)	Advanced NSCLC	No	First	Double	226	Chemo + Erlotinib	150 mg/d	80.50	42.90	10.00	18.30

DCR, disease control rate; ORR, overall response rate; mPFS, median progression-free survival; mOS, median overall survival; NSCLC, non-small cell lung cancer; EGFR-TKI(s), EGFR tyrosine kinase inhibitors; qd, once a day; bid, twice a day; tid, three times a day; d, day;—, not reported; Chemo, chemotherapy.

The publication dates for all the included articles were concentrated from 2005 to 2021, and the target population was patients with advanced NSCLC. Among the studies, 21 (33.3%) were single-arm clinical trials, 42 (66.7%) were double-arm or multi-arm clinical trials, 34 (54.0%) enrolled patients in the first-line treatment stage, and 29 (46.0%) enrolled patients in the late-line or multi-line treatment stage. The sample sizes ranged from 11 to 479. The targeted therapies included 15 targeted drugs (avapritinib, afatinib, icotinib, alectinib. Anlotinib, osimertinib, brigatinib, dacomitinib, erlotinib, ensatinib, gefitinib, crizotinib, linsitinib, ceritinib, and sorafenib). DCRs ranged from 14.9% to 100.0%; ORRs values ranged from 5.3% to 87.0%; mPFS ranged from 1.5 to 20.0 months; and mOS ranged from 3.2 to 34.0 months.

### Evaluation of the Quality of the Included Studies

**FIGURE 2:**
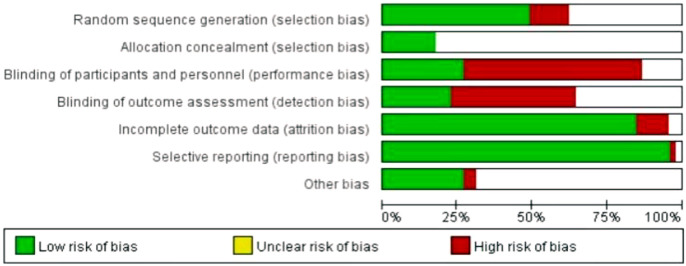
Risk of bias in the included RCTs.

Risk of bias in RCTs: The results of the risk of bias analyses for 42 two-arm or multi-arm RCTs were provided in Figure 2. “Selective reporting,” “incomplete outcome data” and “random sequence generation” had a low risk of bias, and “blinding of outcome assessment” and “blinding of participants and personnel” had a high risk of bias. The risk of bias results for “allocation concealment” and “other bias” were not clear.

**FIGURE 3:**
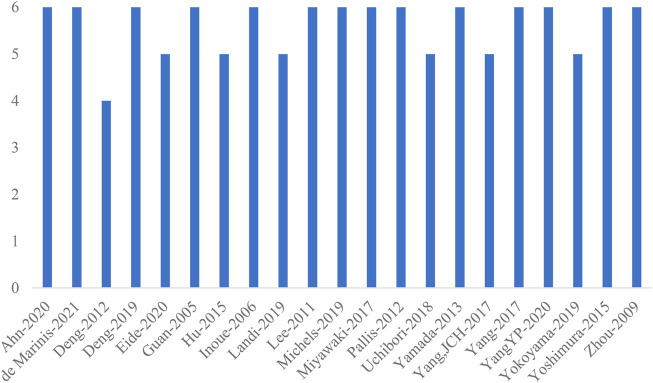
NOS quality scores for the single-arm trials.

Quality evaluation results for single-arm trials: The NOS scores for the 21 included single-arm clinical trials were shown in Figure 3. The NOS scores for all studies ranged from 4 to 6, with an average score of 5.6, indicating that the overall quality of the studies was high. Among them, the NOS score for one paper was four points, the NOS score for six papers was five points, and the NOS score for the remaining 14 papers was six points.

## Relationship Between Short-Term Surrogate Endpoint Indicators and Primary Endpoint Indicators

### Analysis of ORR and mPFS

**FIGURE 4:**
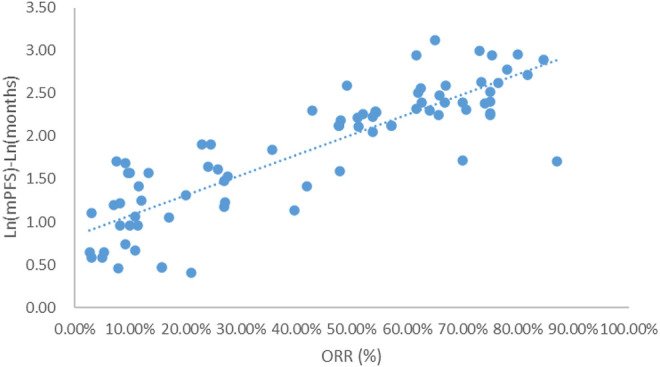
Linear fitting results for ORR and ln (mPFS).

Taking the natural logarithm of mPFS, the adjusted goodness-of-fit of the univariate regression between ORR and ln (mPFS) was excellent (R^2^_adj_ = 0.7356 > 0.6), which was shown in Figure 4 and Supplementary Table S4. After controlling the treatment factors, the adjusted goodness-of-fit of the multivariate regression between ORR and ln (mPFS) was excellent (R^2^_adj_ = 0.7772 > 0.6), which was shown in the Supplementary Table S5.

### Analysis of DCR and mPFS

**FIGURE 5:**
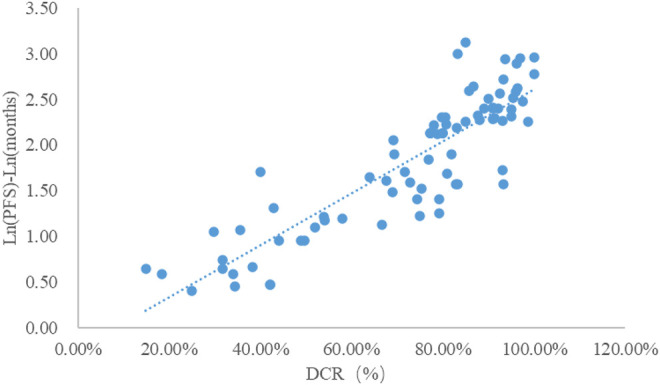
Linear fitting results for DCR and ln (mPFS).

Taking the natural logarithm of mPFS, the adjusted goodness-of-fit of the univariate regression between DCR and ln (mPFS) was excellent (R^2^_adj_ = 0.7642 > 0.6), which was shown in Figure 5 and Supplementary Table S6. After controlling the treatment factors, the adjusted goodness-of-fit of the multivariate regression between DCR and ln (mPFS) was excellent (R^2^_adj_ = 0.7806 > 0.6), which was shown in the Supplementary Table S7.

### Analysis of ORR and mOS

**FIGURE 6:**
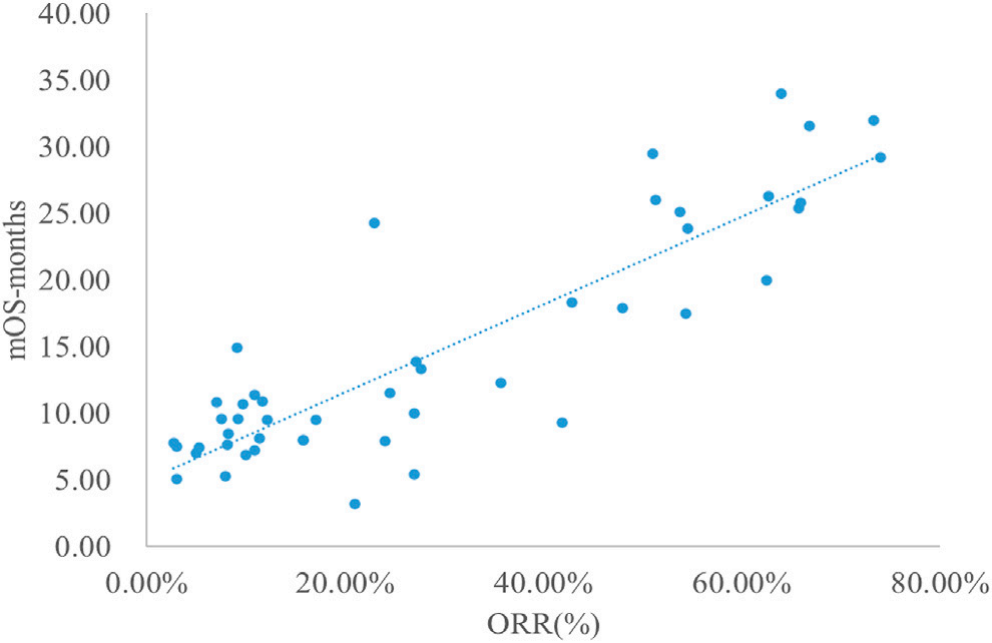
Linear fitting results for ORR and mOS.

The adjusted goodness-of-fit of the univariate regression between ORR and mOS was excellent (R^2^_adj_ = 0.7633 > 0.6), which was shown in Figure 6 and Supplementary Table S8. After controlling the treatment factors, the adjusted goodness-of-fit of the multivariate regression between ORR and mOS was excellent (R^2^_adj_ = 0.7813 > 0.6), which was shown in the Supplementary Table S9.

### Analysis of DCR and mOS

**FIGURE 7:**
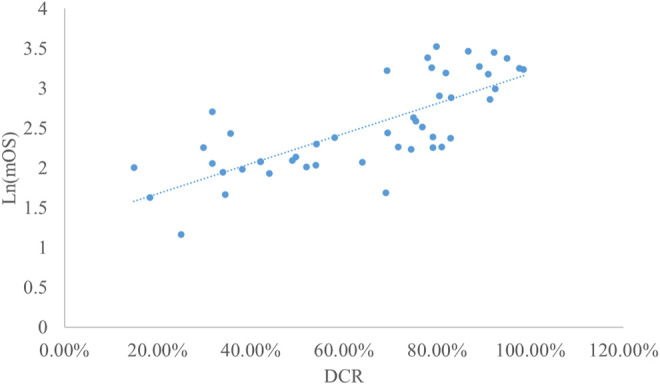
Linear fitting results for DCR and ln (mOS).

Taking the natural logarithm of mOS, the adjusted goodness-of-fit of the univariate regression between DCR and ln (mOS) was good (R^2^_adj_ = 0.5653 > 0.4), which was shown in Figure 7 and Supplementary Table S10. After controlling the treatment factors, the adjusted goodness-of-fit of the multivariate regression between DCR and ln (mOS) was excellent (R^2^_adj_ = 0.6331 > 0.6), which was shown in the Supplementary Table S11.

### Analysis Results of mPFS and mOS

**FIGURE 8:**
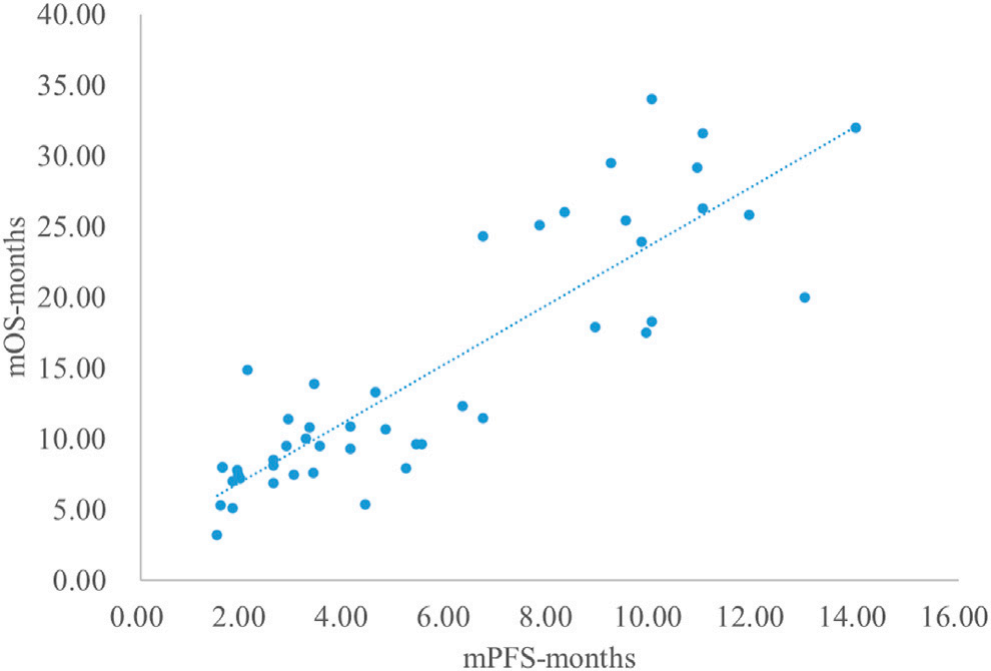
Linear fitting results for mPFS and mOS.

The adjusted goodness-of-fit of the univariate regression between mPFS and mOS was excellent (R^2^_adj_ = 0.7616 > 0.6), which was shown in Figure 8 and Supplementary Table S12. After controlling the treatment factors, the adjusted goodness-of-fit of the multivariate regression between mPFS and mOS was excellent (R^2^_adj_ = 0.8036 > 0.6), which was shown in the Supplementary Table S13.

## Subgroup Analysis

### Results for First-Line Treatment Only

**FIGURE 9:**
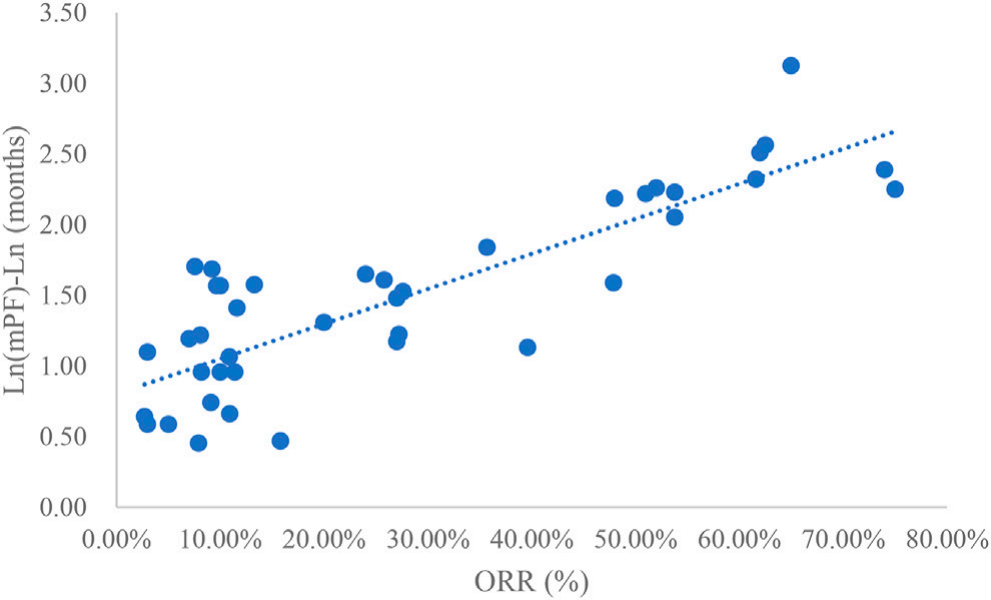
Linear fitting results for ORR and ln (mPFS).

Taking the natural logarithm of mPFS, the adjusted goodness-of-fit of the univariate regression between ORR and ln (mPFS) was excellent (R^2^_adj_ = 0.6188 > 0.6), which was shown in Figure 9.

**FIGURE 10:**
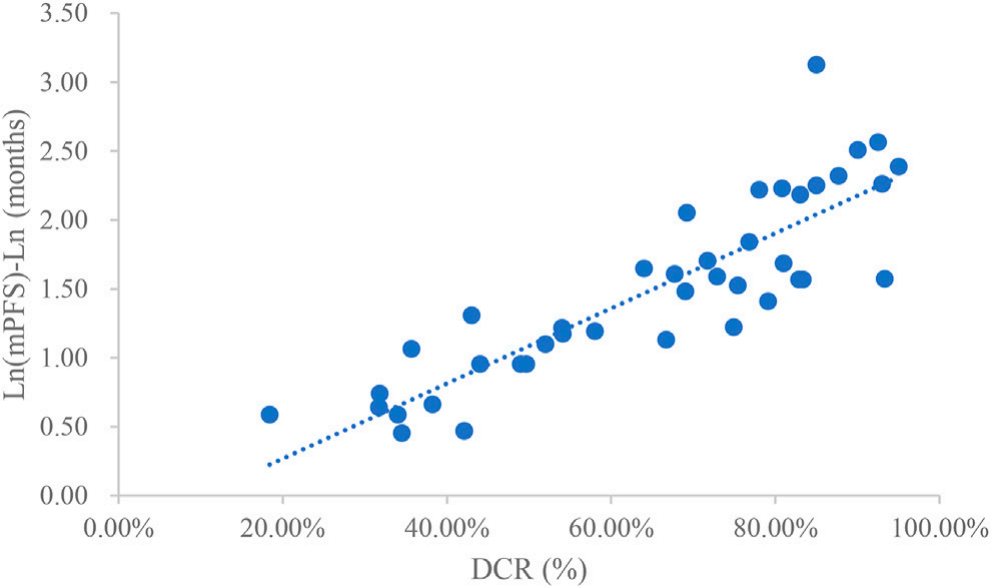
Linear fitting results for DCR and ln (mPFS).

Taking the natural logarithm of mPFS, the adjusted goodness-of-fit of the univariate regression between DCR and ln (mPFS) was excellent (R^2^_adj_ = 0.7128 > 0.6), which was shown in Figure 10.

**FIGURE 11:**
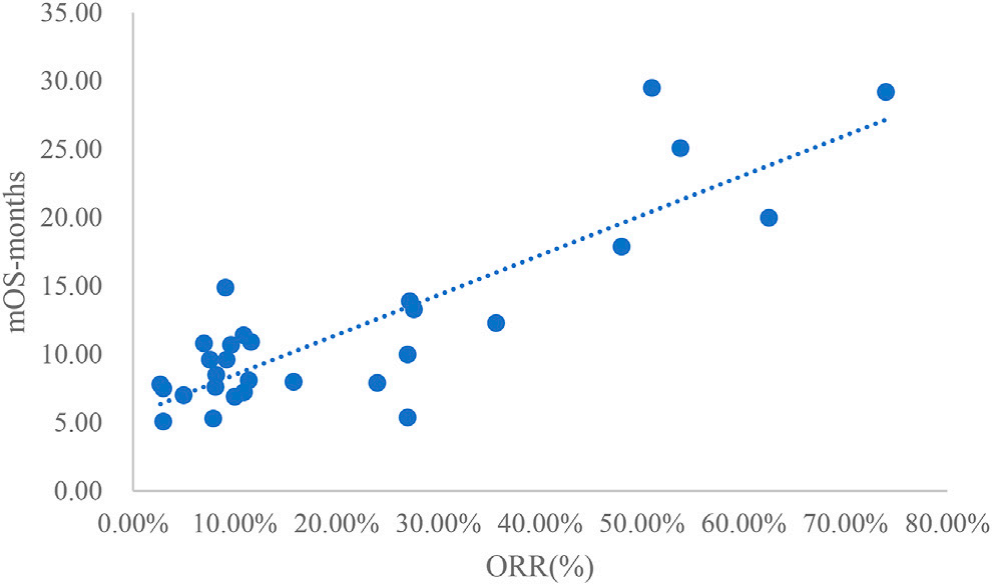
Linear fitting results for ORR and mOS.

The adjusted goodness-of-fit of the univariate regression between ORR and mOS was excellent (R^2^_adj_ = 0.7074 > 0.6), which was shown in Figure 11.

**FIGURE 12:**
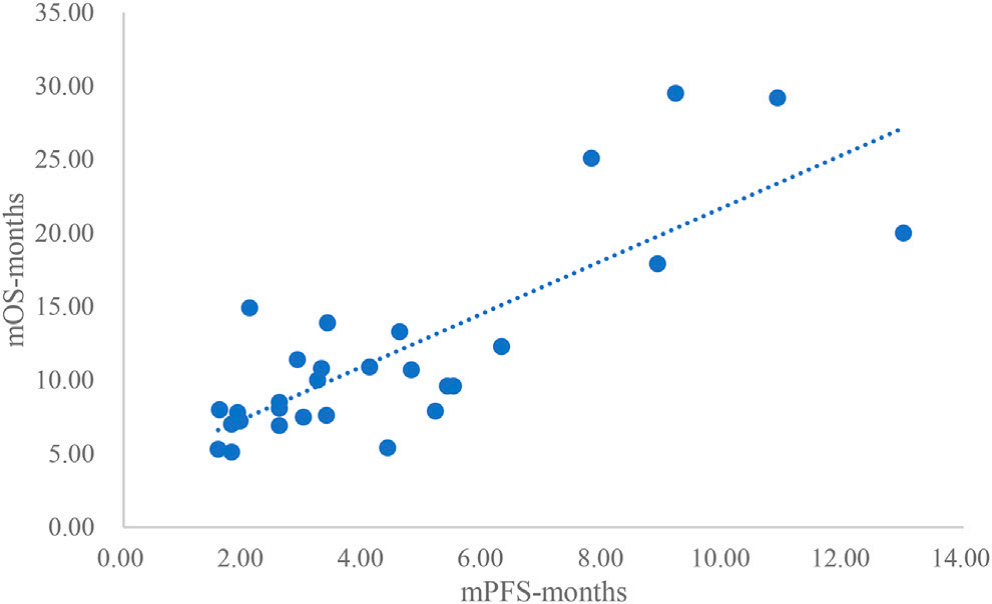
Linear fitting results for mPFS and mOS.

The adjusted goodness-of-fit of the univariate regression between mPFS and mOS was excellent (R^2^_adj_ = 0.7764 > 0.6), which was shown in Figure 12.

### Results of Second-Line or Post-Second-Line Treatment Only

**FIGURE 13:**
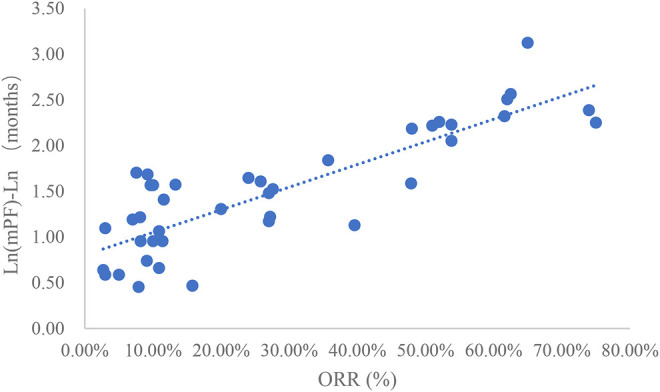
Linear fitting results for ORR and ln (mPFS).

Taking the natural logarithm of mPFS, the adjusted goodness-of-fit of the univariate regression between ORR and ln (mPFS) was excellent (R^2^_adj_ = 0.6926 > 0.6), which was shown in Figure 13.

**FIGURE 14:**
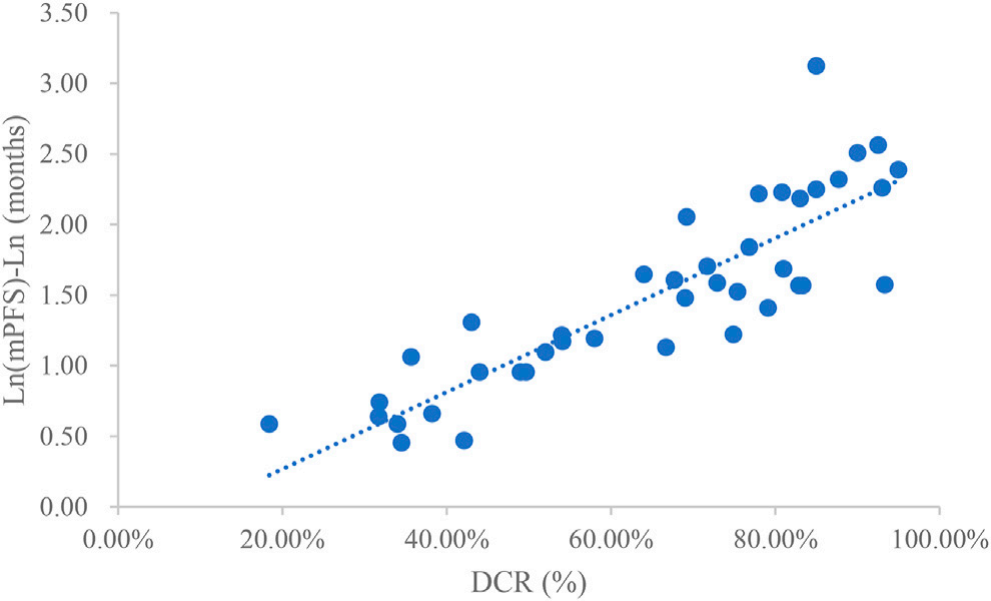
Linear fitting results for ORR and ln (mPFS).

Taking the natural logarithm of mPFS, the adjusted goodness-of-fit of the univariate regression between mPFS and mOS was excellent (R^2^_adj_ = 0.7497 > 0.6), which was shown in Figure 14.

**FIGURE 15:**
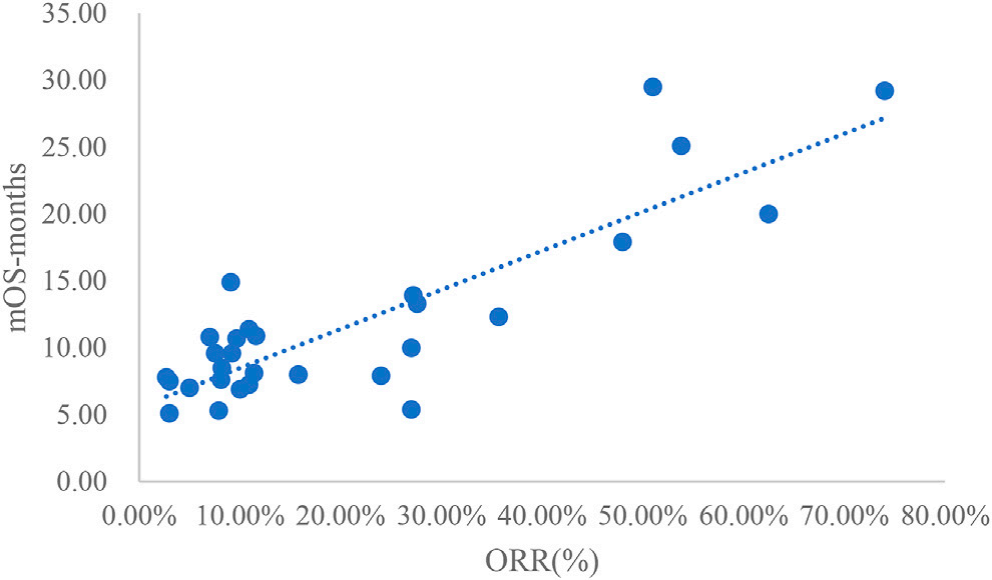
Linear fitting results for ORR and mOS.

The adjusted goodness-of-fit of the univariate regression between ORR and mOS was excellent (R^2^_adj_ = 0.7324 > 0.6), which was shown in Figure 15.

## Discussion

This study summarized all clinical trials of molecular targeted drugs for the treatment of NSCLC approved for marketing in China as of March 2021. Studies that included DCR, ORR, mPFS concurrently and studies that included DCR, ORR, mPFS, and mOS concurrently were extracted for univariate linear regression analysis. This study included a total of 25 articles that reported DCR, ORR, and mPFS concurrently and 39 articles that reported DCR, ORR, mPFS, and mOS concurrently. In the relationship between DCR and ORR and mPFS, DCR and ORR had an excellent linear relationship with ln (mPFS), and the adjusted goodness-of-fit R^2^_adj_ was >0.6. However, the linear relationships between DCR and ORR and mPFS were slightly weaker. For the relationships between DCR and ORR vs. mPFS and mOS, the linear relationship between DCR and mOS or ln (mOS) was good but not excellent (0.4 < R^2^_adj_<0.6). mPFS and mOS had most excellent linear relationships (R^2^_adj_ = 0.8036).

Cooper et al. (2020) (75) conducted a systematic review of the literature that explored the relationship between short-term surrogate endpoint indicators and primary endpoint indicators in malignancy clinical trials. A systematic search of relevant literature in five databases (from the establishment of the database to March 2019) was conducted, resulting in the inclusion of 64 articles. The results of the study suggested that short-term surrogate endpoint indicators such as ORR and CR cannot replace primary endpoint indicators such as PFS and OS and that the correlation between the two is weak and unstable. Our result was inconsistent with those reported by Cooper et al. (Cooper et al., 2020) Although Cooper et al. reported that there was no significant correlation between short-term surrogate endpoint indicators and primary endpoint indicators, the conclusion was likely due to the wide selection of disease types and treatment regimens included in the study. Moreover, Cooper et al. did not address whether there was a correlation between short-term surrogate endpoint indicators and primary endpoint indicators in the clinical trials of specific types of anticancer drugs, which might be one of the main reasons for the difference between the results of this study and the study by Cooper et al.

The results of this study revealed that short-term surrogate endpoint indicators (ORR and DCR) might have a linear relationship with mPFS and mOS, potentially providing the option to use short-term surrogate endpoint indicators to predict mPFS and mOS. In the pharmacoeconomic evaluation of tumors, PFS and OS are the most important evaluation indicators to verify drug efficacy and determine the success of the construction of pharmacoeconomic models. In the traditional pharmacoeconomic models for advanced cancer, 3-state models are often used to construct Markov models or PSMs for pharmacoeconomic evaluations (5). Markov models indicate the transition probability between health states using PFS and OS curves, and PSMs use PFS and OS curves to divide the area under the survival curve into three regions to calculate the area under the curve. If short-term surrogate endpoint indicators are used to predict mPFS and mOS, only two median values can be obtained, posing a challenge for pharmacoeconomic evaluations. We recommend that when only mPFS and mOS are available, assuming the PFS and OS curves obey an exponential distribution, mPFS and mOS should be used to construct an exponential distribution survival curve, thereby allowing the construction of a Markov model and an economic evaluation (Latimer, 2011). Although this method has strong assumptions, it can also provide a certain reference value in the absence of data.

This study has some limitations. Firstly, the molecular targeted drugs included in this study were limited to targeted drugs for the treatment of NSCLC that were approved for marketing in China as of March 2021; many targeted drugs approved for marketing in the other countries were not included in this study. Therefore, the extrapolation of the results is limited. Secondly, although only molecular targeted drugs approved in China were included, the race distribution of the included patients were not considered in the analyses. For many of these drugs, especially the recently approved drugs, were approved based on the published clinical data of the published international population plus the unpublished clinical data of a small sample of the Chinese population. Thirdly, this study did not use a large amount of real-world data for prediction and validation for the focus was to establish the statistical relationship between short-term surrogate endpoint indicators and primary endpoint indicators. Finally, for mOS, in addition to short-term surrogate endpoint indicators, other factors, such as the choice of subsequent treatment, will have a significant impact on mOS; however, the univariate linear regression used in this study did not include enough influencing factors other than treatments.

## Data Availability

The original contributions presented in the study are included in the article/Supplementary Material, further inquiries can be directed to the corresponding author.
