# Supplementary material for: The Relationship Between Short-Term Surrogate Endpoint Indicators and mPFS and mOS in Clinical Trials of Malignant Tumors: A Case Study of Approved Molecular Targeted Drugs for Non-Small-Cell Lung Cancer in China
Source: Front Pharmacol. 2022 Mar 16;13:862640. doi: 10.3389/fphar.2022.862640 (PMC8966682; doi:10.3389/fphar.2022.862640)
Supplement: Supplementary file 1 [file DataSheet1.docx]

**The relationship between short-term surrogate endpoint indicators and mPFS and mOS in clinical trials of malignant tumors: A case study of approved molecular targeted drugs for non-small-cell lung cancer in China**

Mingjun Rui^1,2^, Zijing Wang^1,2^, Zhengyang Fei^1,2^, Yao Wu^1,2^, Yingcheng Wang^1,2^, Lei Sun, Ye Shang^1,2^, Hongchao Li^1,2,*^

^1^ School of International Pharmaceutical Business, China Pharmaceutical University, Nanjing 211198, China;

^2^ Center for Pharmacoeconomics and Outcomes Research, China Pharmaceutical University, Nanjing 211198, China

*Corresponding authors: Hongchao Li, Email: lihongchao@cpu.edu.cn

**Supplementary Materials**

[Supplementary table 1 Pubmed search strategy 1](#_Toc96554966)

[Supplementary table 2 Embase search strategy 2](#_Toc96554967)

[Supplementary table 3 Cochrane Library search strategy 3](#_Toc96554968)

[Supplementary table 4 Univariate regression results for ORR and ln(mPFS) 4](#_Toc96554969)

[Supplementary table 5 Multivariate regression for ORR and ln(mPFS) 5](#_Toc96554970)

[Supplementary table 6 Univariate regression results for DCR and ln(mPFS) 6](#_Toc96554971)

[Supplementary table 7 Multivariate regression for DCR and ln(mPFS) 7](#_Toc96554972)

[Supplementary table 8 Univariate regression for ORR and mOS 8](#_Toc96554973)

[Supplementary table 9 Multivariate regression for ORR and mOS 8](#_Toc96554974)

[Supplementary table 10 Univariate regression for DCR and Ln(mOS) 9](#_Toc96554975)

[Supplementary table 11 Multivariate regression for DCR and ln(mOS) 9](#_Toc96554976)

[Supplementary table 12 Univariate regression for mPFS and mOS 10](#_Toc96554977)

[Supplementary table 13 Multivariate regression for mPFS and mOS 10](#_Toc96554978)

# Supplementary table 1 Pubmed search strategy

| #1 | Non-small Cell Lung Cancer[tiab] OR Nonsmall Cell Lung Cancer[tiab] OR Non-small-cell Lung Cancer[tiab] |
| --- | --- |
| #2 | gefitinib[tiab] |
| #3 | erlotinib[tiab] |
| #4 | Icotinib[tiab] |
| #5 | crizotinib[tiab] |
| #6 | dacomitinib[tiab] |
| #7 | afatinib[tiab] |
| #8 | osimertinib[tiab] |
| #9 | almonertinib[tiab] |
| #10 | alectinib[tiab] |
| #11 | ceritinib[tiab] |
| #12 | brigatinib[tiab] |
| #13 | lorlatinib[tiab] |
| #14 | selpercatinib[tiab] |
| #15 | entrectinib[tiab] |
| #16 | dabrafenib[tiab] |
| #17 | trametinib[tiab] |
| #18 | anlotinib[tiab] |
| #19 | #2 OR #3 OR #4 OR #S OR #6 OR #7 OR #8 OR #9 OR #10 OR #11 OR #12 OR #13 OR #14 OR #15 OR #16 OR #17 OR #18 |
| #20 | clinical stud* [tiab] OR clinical trail*[tiab] OR randomized controlled trial*[tiab] OR trial*[tiab] |
| #21 | #1 AND #19 AND #20 |

# Supplementary table 2 Embase search strategy

| #1 | Non-small Cell Lung Cancer':ab,kw,ti OR 'Nonsmall Cell Lung Cancer':ab,kw,ti OR 'Non-small-cell Lung Cancer':ab,kw,ti |
| --- | --- |
| #2 | gefitinib:ab,kw,ti |
| #3 | erlotinib:ab,kw,ti |
| #4 | Icotinib:ab,kw,ti |
| #5 | crizotinib:ab,kw,ti |
| #6 | dacomitinib:ab,kw,ti |
| #7 | afatinib:ab,kw,ti |
| #8 | osimertinib:ab,kw,ti |
| #9 | almonertinib:ab,kw,ti |
| #10 | alectinib:ab,kw,ti |
| #11 | ceritinib:ab,kw,ti |
| #12 | brigatinib:ab,kw,ti |
| #13 | lorlatinib:ab,kw,ti |
| #14 | selpercatinib:ab,kw,ti |
| #15 | entrectinib:ab,kw,ti |
| #16 | dabrafenib:ab,kw,ti |
| #17 | trametinib:ab,kw,ti |
| #18 | anlotinib:ab,kw,ti |
| #19 | #2 OR #3 OR #4 OR #S OR #6 OR #7 OR #8 OR #9 OR #10 OR #11 OR #12 OR #13 OR #14 OR #15 OR #16 OR #17 OR #18 |
| #20 | clinical stud*':ab,kw,ti OR 'clinical trail*':ab,kw,ti OR 'randomized controlled trial*':ab,kw,ti OR trial*:ab,kw,ti |
| #21 | #1 AND #19 AND #20 |

# Supplementary table 3 Cochrane Library search strategy

| #1 | (Non-small Cell Lung Cancer):ti,ab,kw OR (Nonsmall Cell Lung Cancer):ti,ab,kw OR (Non-small-cell Lung Cancer):ti,ab,kw |
| --- | --- |
| #2 | gefitinib:ti,ab,kw |
| #3 | erlotinib:ti,ab,kw |
| #4 | Icotinib:ti,ab,kw |
| #5 | crizotinib:ti,ab,kw |
| #6 | dacomitinib:ti,ab,kw |
| #7 | afatinib:ti,ab,kw |
| #8 | osimertinib:ti,ab,kw |
| #9 | almonertinib:ti,ab,kw |
| #10 | alectinib:ti,ab,kw |
| #11 | ceritinib:ti,ab,kw |
| #12 | brigatinib:ti,ab,kw |
| #13 | lorlatinib:ti,ab,kw |
| #14 | selpercatinib:ti,ab,kw |
| #15 | entrectinib:ti,ab,kw |
| #16 | dabrafenib:ti,ab,kw |
| #17 | trametinib:ti,ab,kw |
| #18 | anlotinib:ti,ab,kw |
| #19 | #2 OR #3 OR #4 OR #S OR #6 OR #7 OR #8 OR #9 OR #10 OR #11 OR #12 OR #13 OR #14 OR #15 OR #16 OR #17 OR #18 |
| #20 | (clinical stud*):ti,ab,kw OR (clinical trail*):ti,ab,kw OR (randomized controlled trial*):ti,ab,kw OR trial*:ti,ab,kw |
| #21 | #1 AND #19 AND #20 |

# Supplementary table 4 Univariate regression results for ORR and ln(mPFS)


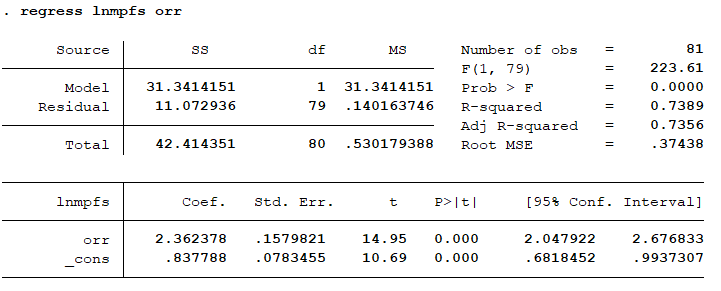


# Supplementary table 5 Multivariate regression for ORR and ln(mPFS)


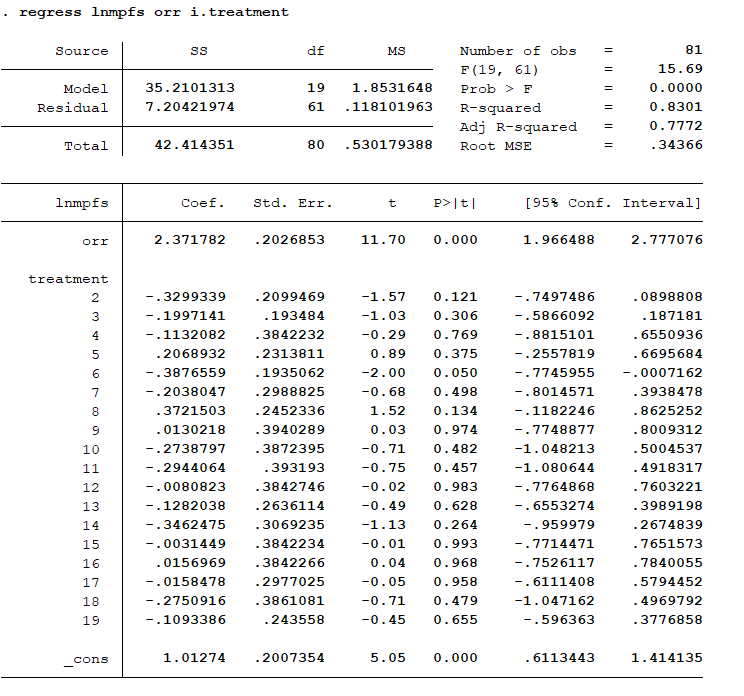


1: afatinib; 2: osimertinib; 3: gefitinib; 4: alectinib; 5: crizotinib; 6: erlotinib; 7: ceritinib; 8: anlotinib; 9: erlotinib + sorafenib; 10: erlotinib + linsitinib; 11: erlotinib + pazotinib; 12: EGFR-TKIs; 13: icotinib; 14: dacomitinib; 15: brigatinib;16: entrectinib; 17: EGFR-TKIs + linsitinib; 18: avapritinib; 19: EGFR-TKIs+chemotherapy

# Supplementary table 6 Univariate regression results for DCR and ln(mPFS)


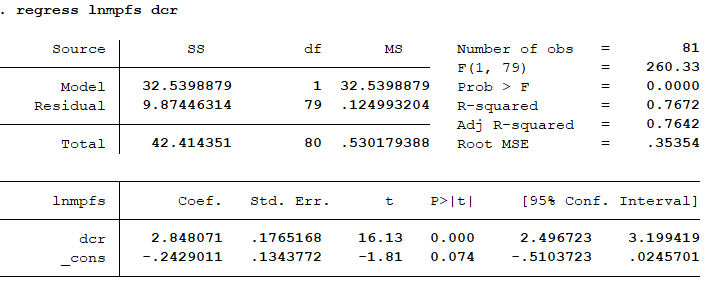


# Supplementary table 7 Multivariate regression for DCR and ln(mPFS)


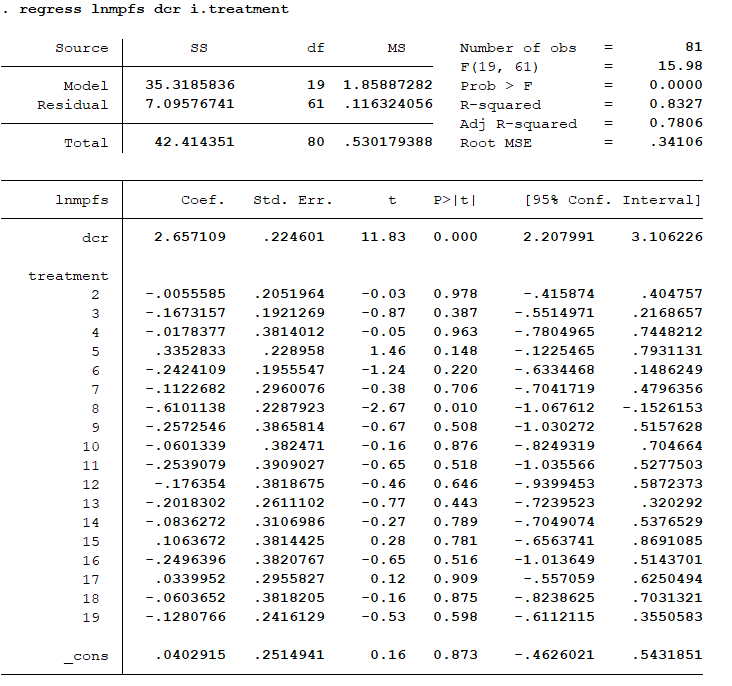


1: afatinib; 2: osimertinib; 3: gefitinib; 4: alectinib; 5: crizotinib; 6: erlotinib; 7: ceritinib; 8: anlotinib; 9: erlotinib + sorafenib; 10: erlotinib + linsitinib; 11: erlotinib + pazotinib; 12: EGFR-TKIs; 13: icotinib; 14: dacomitinib; 15: brigatinib;16: entrectinib; 17: EGFR-TKIs + linsitinib; 18: avapritinib; 19: EGFR-TKIs+chemotherapy

# Supplementary table 8 Univariate regression for ORR and mOS


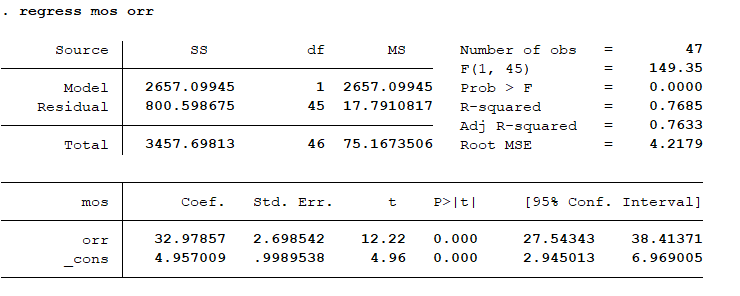


# Supplementary table 9 Multivariate regression for ORR and mOS


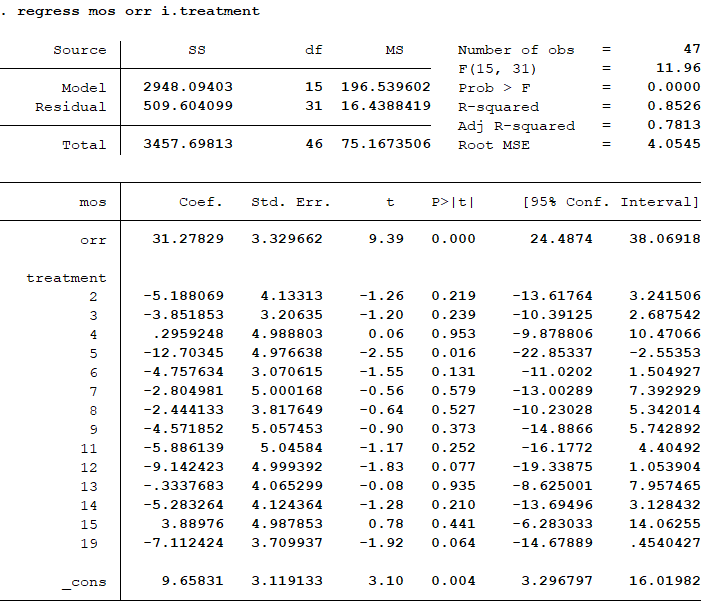


1: afatinib; 2: osimertinib; 3: gefitinib; 4: alectinib; 5: crizotinib; 6: erlotinib; 7: ceritinib; 8: anlotinib; 9: erlotinib + sorafenib; 10: erlotinib + linsitinib; 11: erlotinib + pazotinib; 12: EGFR-TKIs; 13: icotinib; 14: dacomitinib; 15: brigatinib;16: entrectinib; 17: EGFR-TKIs + linsitinib; 18: avapritinib; 19: EGFR-TKIs+chemotherapy

# Supplementary table 10 Univariate regression for DCR and Ln(mOS)


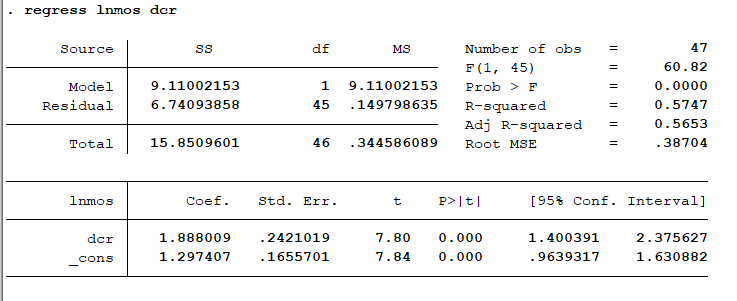


# Supplementary table 11 Multivariate regression for DCR and ln(mOS)


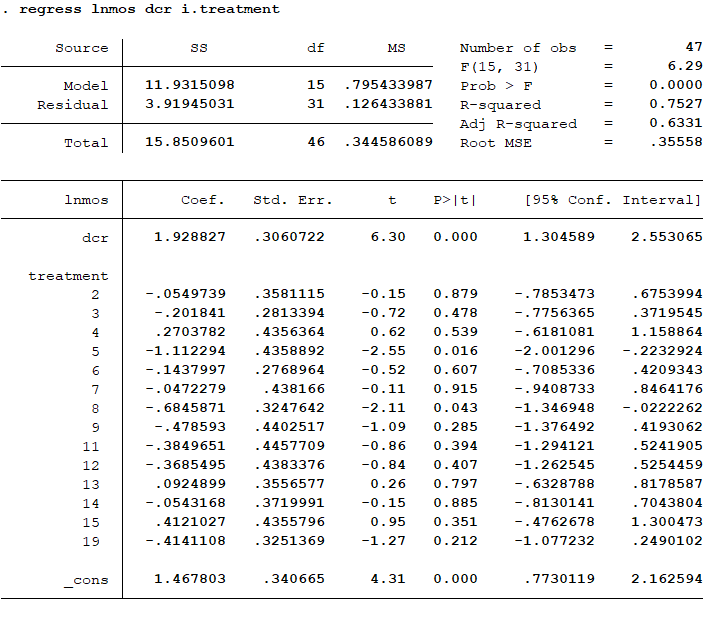


1: afatinib; 2: osimertinib; 3: gefitinib; 4: alectinib; 5: crizotinib; 6: erlotinib; 7: ceritinib; 8: anlotinib; 9: erlotinib + sorafenib; 10: erlotinib + linsitinib; 11: erlotinib + pazotinib; 12: EGFR-TKIs; 13: icotinib; 14: dacomitinib; 15: brigatinib;16: entrectinib; 17: EGFR-TKIs + linsitinib; 18: avapritinib; 19: EGFR-TKIs+chemotherapy

# Supplementary table 12 Univariate regression for mPFS and mOS


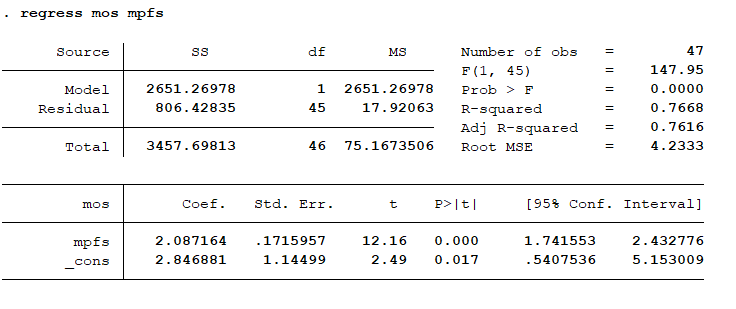


# Supplementary table 13 Multivariate regression for mPFS and mOS


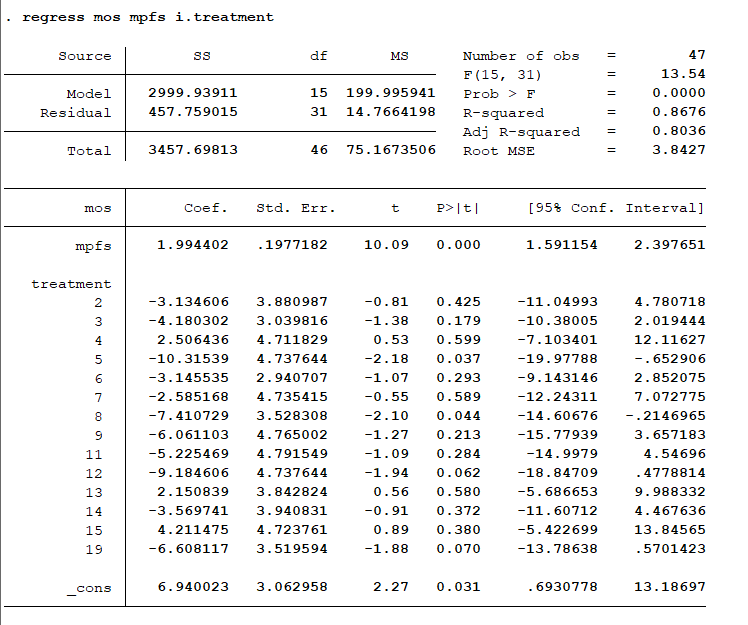


1: afatinib; 2: osimertinib; 3: gefitinib; 4: alectinib; 5: crizotinib; 6: erlotinib; 7: ceritinib; 8: anlotinib; 9: erlotinib + sorafenib; 10: erlotinib + linsitinib; 11: erlotinib + pazotinib; 12: EGFR-TKIs; 13: icotinib; 14: dacomitinib; 15: brigatinib;16: entrectinib; 17: EGFR-TKIs + linsitinib; 18: avapritinib; 19: EGFR-TKIs+chemotherapy
